# Association between shift work and microalbuminuria: data from KNHANES(2012–2014)

**DOI:** 10.1186/s40557-017-0194-8

**Published:** 2017-08-21

**Authors:** Eun Kye Kang, Gu Hyeok Kang, Jun Young Uhm, Young Gon Choi, Soo Young Kim, Seong Sil Chang, Hyoung-Ryoul Kim

**Affiliations:** 1 Department of Occupational and Environmental Medicine, Eulji University Hospital, Daejeon, Republic of Korea; 2 Department of Occupational and Environmental Medicine, College of Medicine, The Catholic University of South Korea, 222 Banpo-Daero, Seocho-Gu, Seoul 137701 Republic of Korea

**Keywords:** Shift work, Microalbuminuria, Cardiovascular disease, KNAHNES

## Abstract

**Background:**

Shift work disturbs workers’ biological clocks and this condition can cause various health problems including cardiovascular disease. The elevated albuminuria level has been significantly associated with the risk of the cardiovascular disease even within a normal reference range. Therefore, this study aimed to investigate the association between shift work and microalbuminuria.

**Methods:**

Workers aged over 20 years from the fifth and sixth Korea National Health and Nutrition Examination Survey(KNHANES 2012–2014; *n* = 3000) were included in this analysis. The multiple logistic regression analysis was performed to determine the association between shift work and microalbuminuria stratified by gender.

**Results:**

The prevalence of microalbuminuria in male subjects was higher among day workers, but the difference was not significant. However, the prevalence of microalbuminuria among females was higher in shift workers with statistical significance. For female, the Odds ratio of microalbuminuria in shift workers was significantly higher with 1.86 (95% CI 1.02–3.39) compared with day workers. After dividing into 5 subgroups of the shift work pattern, the odds ratio of microalbuminuria for fixed night shift was significantly higher at 4.68 (95% CI 1.29–17.00) compared with day workers.

**Conclusions:**

This study showed that shift work was associated with microalbuminuria in female workers. Especially we found out the association between fixed night shift and microalbuminuria in female workers.

## Background

Humans normally work during the day and sleep at night. With the development of modern industries, humans can work at all hours of the day and night [1]. Consequently, so-called ‘shift work,’ which provides labor at unusual times, is unavoidable [2]. To keep society running 24 h a day, shift work has become a form of work that goes against humans’ natural circadian rhythms [3].

Shift work means that the workers are engaged on ‘non-standard’ working hours, including shift and night work, though there are various definitions of shift work [2]. According to the 6th European Working Conditions Survey (2015), the proportion of shift workers in 28 European countries has increased from 17% in 2005 and 2010 to 21% in 2015 [4]. In South Korea, 13.19% of men and 11.63% of women are engaged in night work, and most of those who do shift work are manual workers, according to the 2011 Korea National Health and Nutrition Examination Survey [5].

Unlike day workers, shift workers who work at unusual time will be artificially exposed to light at the wrong times [1]. As a result, their biological clocks become disturbed and when this condition occurs over a prolonged period, it can cause various health problems. Shift work, including night work, is associated with an increased risk of gastrointestinal disease, cardiovascular disease, diabetes, metabolic syndrome, breast cancer, prostate cancer, and sleep-related problems [6–8]. In addition, working at night may affect behavior such as eating habits, social relationships, and smoking ultimately resulting in health problems [9].

Cardiovascular disease, among the various health hazards caused by shift work, is one of the leading causes of death in most industrialized countries, and was estimated to be the cause of 17.5 million deaths worldwide in 2012 [10, 11]. Despite remarkable advances in treatment methods, cardiovascular disease is responsible for a significant disease burden and its prevention should be emphasized. Although the causes of cardiovascular disease are multifactorial, previous cohort studies have demonstrated that shift workers who work the night shift might have an increased risk of cardiovascular disease compared with day workers [12, 13].

Microalbuminuria is an indicator of renal impairment in patients with diabetes or hypertension [14]. Microalbuminuria is also a surrogate marker of endothelial dysfunction, and is recognized as a pre-disease state or risk factor for cardiovascular disease before the onset of cardiovascular disease [15]. The presence of microalbuminuria in normal individuals can be interpreted to mean that the risk of cardiovascular disease is high [16].

There are a number of studies on shift work and cardiovascular disease, but studies dealing with microalbuminuria, a pre-disease marker of cardiovascular disease, are rare. Because cardiovascular events do not frequently occur, microalbuminuria can be used to easily identify the association between shift work and increased risk of cardiovascular disease. In addition, microalbuminuria can help to clarify the mechanism of the development of cardiovascular disease due to shift work.

Despite the importance of microalbuminuria as an independent risk factor for cardiovascular disease, there have been few previous studies on the association between microalbuminuria and shift work. Therefore, the present study aimed to investigate the association between shift work and microalbuminuria, and the prevalence of microalbuminuria according to shift work patterns using data from the Korea National Health and Nutrition Examination Survey.

## Methods

### Subject

Data from the fifth and sixth KNHANES, gathered since 2012 to 2014, was used for the analyses. The KNHANES is a multistage stratified complex design survey of a representative sample of the entire Korean population conducted by the Korea Centers for Disease Control and Prevention. Trained interviewers and laboratory technicians conducted surveys in households, including administering questionnaires, performing health examinations, and collecting blood samples. The total number of KHANES subjects in 2012 to 2014 is 23,626. Among them, 9343 people answered that they have a job, and 8408 people have jobs other than agriculture and fisheries (*n* = 924), and soldiers (*n* = 11). Of the 8408 workers, 4012 manual workers were selected. 3000 participants were selected as final study subjects after excluding CKD (*n* = 6), pregnant (*n* = 9), menstruation (*n* = 772), ACR > 300 mg/dl(*n* = 26), group without blood or urine sample (*n* = 131), subjects aged lower than 20 (*n* = 48), and unresponsive of work pattern question(*n* = 20). Consequently, a total of 3000 subjects were analyzed.

### Operational definition of day work and shift work

In KNHANES, participants who answered “Work mostly during day time (between 6 a.m. ~ 6 p.m.)” were classified as day workers, while the rest of the participants who answered “Fixed evening shift (between 2 p.m. ~ midnight), fixed night shift (between 9 p.m. ~ 8 a.m.), regular day and night rotating shift, 24-hours rotating shift, split shift (working two shift in one day), irregular rotating shift” were classified as shift workers. The work schedule was subdivided into 5 groups of day works, fixed night shift, fixed evening shift, 24-h rotating shift, and regular rotating 2 or 3 shifts.

### Assessment and definition of microalbuminuria

A random urine sample was collected during the first morning void, and urinary albumin concentration (μg) was measured using a turbidimetric immunoassay (Hitachi Automatic Analyzer 7600). Urinary creatinine concentration (mg) was measured using a colorimetric method (Hitachi Automatic Analyzer 7600), and the albumin-to-creatinine ratio (mg/g) was calculated by dividing the urinary albumin concentration by the urinary creatinine concentration. Microalbuminuria was defined as a urinary albumin-to-creatinine ratio of 30 to 300 mg/g, in accordance with the definition of the Kidney Disease Improving Global Outcomes(KDIGO) [14].

### Covariates

Age, level of education, household income, smoking status, high-risk drinking behavior, body mass index, systolic blood pressure, diastolic blood pressure, triglyceride, fasting glucose and estimated glomerular filtration rate were included as covariates in this study. Information regarding demographic and social factors was obtained using a standardized questionnaire during the health interview. The levels of education were categorized into elementary school graduates (6-year course), middle school graduates (3-year), high school graduates (3-year), and some university education or above (2 or more years). Household income was classified by quartile as low, low-middle, middle-high, or high levels. Smoking status was divided into non-smokers, ex-smokers, and current smokers. Heavy alcohol drinking was categorized as drinking four or more times per week and 3 units of alcohol per time. After the height and weight were measured using standardized techniques and equipment, body mass index (BMI) was calculated by dividing body weight by height squared (kg/m^2^). Blood pressure (BP) was measured by standard methods using a sphygmomanometer with the patient in a sitting position. After fasting for at least 8 h, blood samples were collected in the morning and analyzed at a central, certified laboratory. Estimated glomerular filtration rate was calculated using the Modification of Diet in Renal Disease formula. The MDRD is (ml/min/1.73m^2^) = 175×(Scr/88.4^)-1.154^xAge^-0.203^ × 0.742 (if, female).

### Statistical analyses

The statistical analyses were performed using SPSS version 18.0 to take into account sample weights and complex sample design effects. Since KHNANES uses a complex sampling design, the missing values were set to valid values to analyze statistically. Chi-squared test was used to compare the prevalence of the microalbuminuria according to the categorical variables. Odds ratio was calculated by multiple logistic regression analysis to determine the association between work schedule and microalbuminuria after stratification for gender. The multiple logistic regression analysis was performed, adjusting for age, body mass index, systolic blood pressure, fasting glucose, history of hypertension, history of diabetes, income, educational status, working hours for a week..

### Ethics statement

The present study was approved by the Institutional Review Board of College of Medicine, The Catholic University of Korea(IRB number: 2017–3569-0001).

## Results

### General characteristics of the participants

The present study involved a total of 3000 subjects, consisting of 2094 (79.8%) male subjects and 906 (20.2%) female subjects. The mean age of the participants was 48 years. The mean age of the male subjects and female subjects were 45 and 59 years, respectively. The general characteristics of the subjects are shown in Table 1. In terms of work schedule, the proportion of day work was higher for both male and female workers, and was slightly higher for female workers. In terms of education level, the proportion of high school graduates was higher for males, whereas the proportion of those with elementary school education or below was higher for females. Among lifestyle factors, the smoking rate was high among men and the rate of excessive drinking was low for both male and female subjects.

**Table 1:** General characteristics of the subjects

Variables		Male	Female	Total
Total (N^a^)		2094	906	3000
Age (years)	20–29	148 (12.1%)	1 (0.3%)	149 (9.7%)
	30–39	404 (22.1%)	3 (0.2%)	407 (17.7%)
	40–49	456 (25.7%)	36 (5.2%)	492 (21.5%)
	50–59	567 (26.9%)	401 (50.5%)	968 (31.7%)
	≥ 60	519 (13.2%)	465 (43.8%)	984 (19.4%)
Body mass index(kg/m^2^)	<23	740 (36.5%)	326 (37.7%)	1066 (36.7%)
(*n* = 2988)	23–25	519 (24.8%)	258 (27.6%)	777 (25.4%)
	≥25	824 (38.7%)	321 (34.7%)	1145 (37.9%)
Education	≤ Elementary school	349 (12.3%)	485 (50.5%)	834 (20.0%)
(*n* = 2999)	Middle school	330 (13.9%)	187 (22.7%)	517 (15.7%)
	High school	939 (49.3%)	200 (22.9%)	1139 (44.0%)
	≥ University	475 (24.5%)	34 (3.9%)	509 (20.3%)
Household income	Low	533 (26.9%)	192 (21.9%)	725 (25.9%)
(*n* = 2979)	Low-middle	606 (28.8%)	296 (32.3%)	902 (29.5%)
	Middle-high	542 (25.5%)	241 (26.0%)	783 (25.6%)
	High	397 (18.8%)	172 (19.8%)	569 (19.0%)
Work pattern	Day-worker	1653 (80.0%)	792 (86.8%)	2445 (81.4%)
	Shift worker	441 (20.0%)	114 (13.2%)	555 (18.6%)
Smoking status	Non-smoker	358 (17.8%)	843 (94.2%)	1201 (33.2%)
(*n* = 2985)	Ex-smoker	752 (32.5%)	24 (2.6%)	776 (26.5%)
	Current smoker	977 (49.7%)	31 (3.2%)	1008 (40.4%)
eavy alcohol drink	Yes	280 (12.7%)	8 (1.1%)	288 (10.4%)
(*n* = 2985)	No	1806 (87.3%)	891 (98.9%)	2697 (89.6%)
History of hypertension	Yes	682 (28.0%)	372 (39.3%)	1054 (30.3%)
(*n* = 2987)	No	1403 (72.0%)	530 (60.7%)	1933 (69.7%)
History of diabetes mellitus	Yes	235 (9.4%)	103 (10.5%)	338 (9.6%)
(*n* = 2870)	No	1775 (90.6%)	757 (89.5%)	2532 (90.4%)
Systolic blood pressure (mmHg)		119.62 ± 0.39	124.08 ± 0.74	120.52 ± 0.37
Diastolic blood pressure (mmHg)		78.99 ± 0.29	76.17 ± 0.45	78.42 ± 0.26
Fasting blood sugar (mg/dl)		100.15 ± 0.47	99.90 ± 0.93	100.10 ± 0.44
Triglyceride (mg/dl)		162.20 ± 3.40	124.10 ± 2.75	154.64 ± 2.81
Estimated glomerular filtration rate		95.28 ± 0.36	95.52 ± 0.32	95.33 ± 0.30

^a^unweighted count

Data are shown as N^a^ (estimated percentage) for categorical variables and as mean ± standard error for continuous variables

### Prevalence of microalbuminuria according to work schedules

The subjects were divided into two groups, the normal and microalbuminuria groups (based on an albumin to creatinine ratio of 30) and the prevalence of microalbuminuria for each variable was investigated. Among male subjects, the prevalence of microalbuminuria increased with age, whereas such a pattern was not found for women. However, the prevalence of microalbuminuria was the highest in those aged 60 years or older regardless of gender. In terms of the prevalence of microalbuminuria by work schedules, the prevalence of microalbuminuria in male subjects was higher among day workers, but the difference was not significant. The prevalence of microalbuminuria among females was higher in shift workers, a difference that was statistically significant. When the participants were divided into three groups of normal, overweight, and obese based on BMI, the prevalence of microalbuminuria was the highest in the obese group for both male and female, a difference that was statistically significant among male, but was not significant among female. There were no statistically significant differences in the prevalence of microalbuminuria according to education level, income, smoking status, or drinking status (Table 2).

**Table 2:** Prevalence of microalbuminuria according to variables

Variables		Male			Female		
		ACR < 30	ACR ≥ 30	P-value	ACR < 30	ACR ≥ 30	*P*-value
Age (years)	20–29	99.5(0.5)	0.5(0.5)	<0.01		100(0.0)	<0.01
	30–39	95.6(1.2)	4.4(1.2)		100(0.0)		
	40–49	95.0(1.2)	5.0(1.2)		92.3(4.3)	7.7(4.3)	
	50–59	93.4(1.2)	6.6(1.2)		92.8(1.4)	7.2(1.4)	
	≥ 60	89.0(1.7)	11.0(1.7)		86.4(2.0)	13.6(2.0)	
Body mass index	<23	95.7(0.9)	4.3(0.9)	<0.01	93.1(1.8)	6.9(1.8)	0.07
	23–25	97.1(0.7)	2.9(0.7)		89.3(2.2)	10.7(2.2)	
	≥25	91.5(1.0)	8.5 (1.0)		86.4(2.0)	13.6(2.0)	
Education	≤ Elementary school	92.4(1.6)	7.6(1.6)	0.30	87.5(1.7)	12.5(1.7)	0.23
	Middle school	93.9(1.6)	6.1(1.6)		93.6(1.9)	6.4(1.9)	
	High school	94.4(0.8)	5.6(0.8)		90.2(2.7)	9.8(2.7)	
	≥ University	95.9(1.0)	4.1(1.0)		94.5(5.3)	5.5(5.3)	
Household income	Low	95.4(1.0)	4.6(1.0)	0.44	90.0(2.3)	10.0(2.3)	0.63
	Low-middle	93.0(1.2)	7.0(1.2)		90.5(1.9)	9.5(1.9)	
	Middle-high	94.7(1.0)	5.3(1.0)		87.3(2.4)	12.7(2.4)	
	High	94.7(1.4)	5.3(1.4)		91.4(2.8)	8.6(2.8)	
Work pattern	Day-worker	94.2(0.7)	5.8(0.7)	0.34	90.8(1.2)	9.2(1.2)	<0.05
	Shift worker	95.4(1.0)	4.6(1.0)		83.1(3.8)	16.9(3.8)	
Smoking status	Non-smoker	95.2(1.3)	4.8(1.3)	0.67	90.0(1.2)	10.0(1.2)	0.28
	Ex-smoker	94.9(1.0)	5.1(1.0)		79.0(9.1)	21.0(9.1)	
	Current smoker	93.9(0.9)	6.1(0.9)		89.1(6.3)	10.9(6.3)	
Heavy alcohol drink	Yes	91.9(1.7)	8.1(1.7)	0.06	95.6(4.6)	4.4(4.6)	0.38
	No	94.8(0.6)	5.2(0.6)		89.6(1.1)	10.4(1.1)	

Data are shown as percentage(standard error) for categorical variables

*P*-value is calculated by χ^2^ test for categorical variables

### Odds ratios of microalbuminuria according to work patterns

Table 3 shows the results of multiple logistic regression analyses, in which the odds ratios (ORs) of microalbuminuria according to work patterns are presented. For male, the OR of microalbuminuria in male shift workers was found to be low, but the difference was not significant. For female, the OR of microalbuminuria in female shift workers was significantly higher with OR 1.86 (95% CI 1.02–3.39) compared with female day workers. After adjusting for age, BMI, systolic blood pressure, fasting blood glucose, triglycerides, glomerular filtration rate, hypertension, the presence or absence of diabetes mellitus, and lifestyle factors, the same results were obtained. After additionally adjusting for income, education status, and working hours per week, the same results were obtained (Table 3). The work patterns was subdivided into 5 groups of day work, fixed night shift, fixed evening shift, 24-h shift work, and regular rotating 2 or 3 shifts (Table 4). For male, the ORs of microalbuminuria for various patterns of shift work in relation to day work were all not significant. For female, the ORs of microalbuminuria for regular rotating 2 or 3 shifts and fixed evening shift in relation to day work were not significant, but the OR of microalbuminuria for fixed night shift compare with day workers was significantly higher at 4.68 (95% CI 1.29–17.00). Even after adjusting for the various variables presented in Table 3, similar results were obtained.

**Table 3:** Crude and adjusted odds ratio for microalbuminuria by work pattern in male and female subjects

	Work pattern	Manual workers		
		Crude OR (95% CI)	Model 1	Model 2
male	Day work	Reference	Reference	Reference
(*n* = 2094)	Shift work	0.74 (0.45–1.20)	0.77 (0.45–1.34)	0.78 (0.46–1.31)
female	Day work	Reference	Reference	Reference
(*n* = 906)	Shift work	1.86 (1.02–3.39)	2.28 (1.12–4.64)	2.29 (1.14–4.60)

OR, odds ratio; CI, confidence interval. Model 1. Adjusted for age, body mass index, systolic blood pressure, fasting glucose, triglyceride, estimated glomerular filtration rate, history of hypertension, history of diabetes, smoking, alcohol. Model 2. Adjusted for age, body mass index, systolic blood pressure, fasting glucose, triglyceride, estimated glomerular filtration rate, history of hypertension, history of diabetes, smoking, alcohol, income, educational status, work hours for a week

**Table 4:** Crude and adjusted odds ratio for microalbuminuria by shift work patterns in male and female subjects

	Shift work patterns	Manual workers		
		Crude OR (95% CI)	Model 1	Model 2
male	Day work	Reference	Reference	Reference
(*n* = 2094)	24-h shift	1.21 (0.49–3.02)	0.85 (0.31–2.37)	0.90 (0.31–2.63)
	Rotating 2 or 3 shifts	0.65 (0.22–1.93)	0.63 (0.17–2.33)	0.61 (0.17–2.22)
	Fixed evening	0.58 (0.23–1.48)	0.69 (0.24–2.00)	0.69 (0.23–2.08)
	Fixed night	0.55 (0.16–1.95)	0.65 (0.18–2.42)	0.60 (0.17–2.19)
female	Day work	Reference	Reference	Reference
(*n* = 906)	Rotating 2 or 3 shifts	1.45 (0.17–12.40)	1.15 (0.06–20.47)	2.03 (0.11–38.71)
	Fixed evening	1.93 (0.97–3.84)	2.01 (0.89–4.51)	1.85 (0.84–4.10)
	Fixed night	4.68 (1.29–17.00)	11.54 (2.65–50.35)	14.60 (3.50–61.01)

OR, odds ratio; CI, confidence interval. Model 1. Adjusted for age, body mass index, systolic blood pressure, fasting glucose, triglyceride, estimated glomerular filtration rate, history of hypertension, history of diabetes, smoking, alcohol. Model 2. Adjusted for age, body mass index, systolic blood pressure, fasting glucose, triglyceride, estimated glomerular filtration rate, history of hypertension, history of diabetes, smoking, alcohol, income, educational status, work hours for a week

## Discussion

The present study aimed to investigate the association between shift work and microalbuminuria. Gender differences on the association between shift work and microalbuminuria was shown in this study. Compared to day work, shift work including fixed night shift was associated with microalbuminuria in female workers. When patterns of shift work were divided into 5 groups, fixed-night shift was significantly associated with microalbuminuria even after adjusting for the confounding variables. However, there was no association between shift work and microalbuminuria among male workers.

Many previous studies have shown that shift work, including the night shift, affects the risk of cardiovascular disease. A 12-year prospective cohort study by Tuchesn et al. examined the association between shift work and cardiovascular disease and found that the relative risk for cardiovascular diseases in shift workers was significant with OR 1.31 (95% CI 1.06–1.63) compared to day workers [17]. In the Danish study, it was found that when the relative risk of being admitted to hospital because of ischemic heart disease (IHD) was compared between shift workers and day workers, shift work and night work were associated with an increased risk of IHD [18]. A 12-year follow-up study by Fujino et al. showed that compared with day workers, shift workers had a significantly higher risk of death due to IHD (relative risk: 2.23, 95% CI 1.37–3.95) and fixed-night shift workers also had a higher but insignificant risk (relative risk: 1.23, 95% CI 0.49–3.10) [19]. In addition, according to two recent meta-analyses of shift work and cardiovascular disease, the risks of hypertension, diabetes, and dyslipidemia or major risk factors for cardiovascular disease were higher in shift workers and the risk of developing IHD linked to death was 1.4 times higher in shift workers compared with day workers [12, 13]. Evidence that shift work is associated with cardiovascular disease is accumulating.

Despite previous studies indicating that night work and shift work have effects on cardiovascular disease, there have been few studies on the association between shift work and microalbuminuria as a pre-disease indicator of cardiovascular disease. Among previous studies, Boogaard and Caubo suggested a relationship between circadian disruption and microalbuminuria. In that study, the subjects were divided into three groups, a group of shift workers exposed to low concentrations of potentially nephrotoxic chemicals, a group of non-exposed shift workers, and a group of non-exposed day workers, and they investigated differences in albuminuria. They found significantly lower urinary albumin concentrations in day workers regardless of exposure to chemicals compared with shift workers, and the authors suggested that their findings were related to circadian rhythms [20]. Previous studies have shown night work and shift work reduce sleep duration and quality [1]. According to a retrospective cohort study by Yamamoto et al., in which the association between self-reported sleep duration and proteinuria measured by the use of a dipstick test was assessed in 2773 night workers and 4061 day workers, a sleep duration of 5 or fewer hours was a predictor of proteinuria compared with those sleeping 7 h per night [21]. Similarly, in a prospective study by CJ Mcmullan et al. of middle-aged women over a 11-year period using data from the Nurse’s health study, a shorter sleep duration was found to be associated with an increased prevalence of albuminuria and a significant decline in renal function (albuminuria: adjusted OR = 2.52; 95% CI 1.42–4.4 and GFR: age-adjusted OR = 1.91, 95% CI 1.27–2.88) [22]. The authors concluded that shift work, including night work, was associated with a decline in renal function.

A disturbed circadian rhythm is the most important factor in the association between shift work and cardiovascular disease. The exact mechanism by which shift work, including night work, causes cardiovascular disease has not been clearly elucidated. However, Puttonen et al. mentioned the term ‘circadian stress’ that induces pathways in which disturbances of circadian rhythm affect individuals in a complex way to cause cardiovascular disease. Because cardiovascular organs are affected by a 24-h circadian rhythm, circadian stress plays an important role in developing diseases [9]. The circadian rhythms, which are regulated by the central clocks in the suprachiasmatic nucleus (SCN) of the hypothalamus, affect major organs such as the heart and kidney. Also, peripheral cardiovascular cells including vascular endothelial cells and vascular smooth cells have their own biological clock system [23].

Although there is no definite theory, the development of microalbuminuria due to the disturbances of circadian rhythms can be explained by non-dipping phenomena and direct damage to vascular endothelial cells in the kidney. The disturbances of circadian rhythms blur the nocturnal blood pressure fall that should occur normally during sleep. Individuals whose nocturnal pressure fall does not normally occur and have a nocturnal pressure fall of <10% are called non-dippers. Because the kidney itself is affected by circadian rhythms, non-dippers have increased levels of aldosterone and an increased tone of the autonomic nervous system. Subsequently, the reabsorption of sodium results in elevated blood pressure and blunted dipping of blood pressure [24]. Non-dippers have an increased incidence of left ventricle hypertrophy, atherosclerotic plaques, and microalbuminuria. It is thought that the non-dipping phenomenon causes target-organ damage to the cardiovascular system and induces the permeability of vascular endothelial cells, resulting in microalbuminuria [23]. Tsiofis et al. investigated the association between microalbuminuria and non-dipping among patients who were newly diagnosed with hypertension and found that the incidence of microalbuminuria was higher in non-dippers than in dippers. The results of multivariate analysis of participants with normal blood pressure levels and without other diseases showed that non-dipping status was positively correlated with microalbuminuria (coefficient = 0.27, *p* = 0.02) [25].

Another hypothesis is that disturbances of circadian rhythm cause direct endothelial cell damage. Because microalbuminuria is a surrogate marker for assessing glomerular function and represents systemic vascular damage, including damage to vascular endothelial cells, it manifests as dysfunction of endothelial cells [26]. In a study by Charles et al., shift work was associated with decreased renal function among shift workers compared with day workers [27]. In addition, an animal study showed that proteinuria was observed in hamsters with SCN removal, suggesting the possibility of direct renal tissue damage. Therefore, a disruption of circadian rhythm may affect endothelial cell function [28]. Night work and shift work cause disturbances of the circadian rhythm and are thus associated with the development of microalbuminuria.

Previous studies analyzing the prevalence of microalbuminuria by work schedules are rare. In a study by Camille et al., based on the third National Health and Nutrition Examination Survey in the US, the prevalence of microalbuminuria in the total population aged 6 years or older was 7.8% [29]. According to the data from the fifth Korea National Health and Nutrition Examination Survey (2011) in Republic of Korea, the prevalence of microalbuminuria in the Korean population aged 19 years or older was 5.2% [30]. The results of the current study showed that the prevalence of microalbuminuria was 5.8% in male day workers and 4.6% in male shift workers, and 9.2% in female day workers and 16.9% in female shift workers. The difference in the prevalence of microalbuminuria between this study and previous studies is thought to be due to differences in the characteristics of the subjects between studies. However, the results of this study were similar to the results of previous studies showing that the prevalence of microalbuminuria was significantly increased with age.

In our study, we expected that shift work, which disturbs circadian rhythms, would be associated with microalbuminuria, and the results showed a significant association between shift work and microalbuminuria among female workers. However, no significant association was found between shift work and microalbuminuria among male workers. There are some speculations about such a gender difference. As they age, females are at increased risk for cardiovascular disease due to their physical characteristics. Differences in metabolism compared with male and hormonal effects due to menopause can further increase the risk of cardiovascular disease among older female shift workers [31]. In addition, female workers have a greater risk of work-related stress and ‘burnout’ compared with male workers. This is because female workers play a double role in work and at home [32, 33]. Hallman et al. suggested gender difference were the result of women being more strongly affected than male workers by psychological factors including burnout, which is predicted to be a risk factor for cardiovascular diseases [34]. Also, A. Leclecr mentioned that male and female in shift work do not perform similar activities and health effects of shift work and pathways may be different in male and female [35].

This study showed that there was a significant association between fixed night work among shift work patterns and microalbuminuria. There is a lot of disagreement about the health hazards of fixed night work unlike rotating shift work. It is assumed that the human circadian rhythm becomes reversely adjusted to night work when workers are continuously engaged in night work [36]. However, in a literature review by Simon, they concluded that <3% of fixed night workers had adjusted to the day-night reversal, as measured by melatonin release patterns. Fixed night workers are supposed to continuously work in unusual environments compared to rotating shift workers [37]. Unusual environments become a threat to our health due to continued environmental stress, affecting blood pressure, body temperature, and the secretion of hormones. Biggi et al. reported that the incidence of metabolic syndrome, which affects the cardiovascular system, tended to be higher in fixed night workers and stressed the circadian metabolic rhythms more than shift work [38]. When shift work was subdivided and analyzed in the present study, the results showed significant results in fixed night shift workers, which supported the results of previous studies.

This study has several limitations. First, the present study used nationally representative data from the Korea National Health and Nutrition Examination Survey (KNHANES), but it is difficult to establish a clear causal relationship between microalbuminuria and night work due to the limitations of a cross-sectional study. Second, the gold standard for measuring microalbuminuria is to collect urine for 24 h to measure proteinuria. However, urine samples were collected at only one time to measure microalbuminuria in this study. It is possible that a 24-h urine collection may be less reliable due to participants’ being inconvenienced, leading to inaccuracies, and there have been previous studies indicating that a single morning urine measurement is as reliable as microalbuminuria measured by a 24-h urine collection [39]. Finally, we failed to correct all of the various factors (e.g. drug, kidney disease, infection status, muscle mass) that could affect microalbuminuria.

Despite these limitations, this study supports an association between work schedules and microalbuminuria which is a predictor of cardiovascular disease, while using nationally representative data in Republic of Korea. The results of the present study found that female shift workers need to be concerned about an increased risk of cardiovascular disease, and, in particular, working only at night shift is significantly associated with an increased risk of cardiovascular disease. We believe the results suggest valuable insights in point of views of public health as well as environmental, biological and clinical understanding of shift work and microalbuminuria. Future programs to prevent cardiovascular disease and well-designed studies to explore this issue further are needed in the future.

## Conclusion

Microalbuminuria is a strong predictor of future risk of cardiovascular disease among healthy adults without diabetes and hypertension. In addition, shift work is a pattern of work that is likely to increase the incidence of cardiovascular disease. Although microalbuminuria does not directly affect cardiovascular disease, those with a microalbuminuria are likely to be vulnerable to cardiovascular disease. Although microalbuminuria has not been extensively used as a predictor of cardiovascular disease, it is valuable because its measurement methods are simple and useful. Female shift workers are at higher risk of cardiovascular disease when they are engaged in fixed night shift and they are vulnerable group in a view of occupational health. Social concerns and plans are considered necessary to protect their health.
